# *In vitro* effects of dual wavelength photobiomodulation on monocytic response in painful temporomandibular disorder

**DOI:** 10.22514/jofph.2025.082

**Published:** 2025-12-12

**Authors:** Maria Gabriela Rodriguez, Mathew Warchol, Selenia Rubio, Patricia Cabrera, Rory Reever, Sherif Hosney, Cesar A. Migliorati, Frank C. Gibson III, Richard Ohrbach, Roger B. Fillingim, Shannon Wallet, Margarete C. Ribeiro Dasilva

**Affiliations:** ^1^Division of Prosthodontics, Restorative Dental Sciences Department, University of Florida College of Dentistry, Gainesville, FL 32610, USA; ^2^Department of Oral Biology, College of Dentistry, University of Florida, Gainesville, FL 32610, USA; ^3^Community Department of Community Dentistry and Behavioral Science, College of Dentistry, University of Florida, Gainesville, FL 32610, USA; ^4^Pain Research and Intervention Center (PRICE), College of Dentistry, University of Florida, Gainesville, FL 32611, USA; ^5^Department of Oral and Maxillofacial Diagnostic Sciences, University of Florida, Gainesville, FL 32610, USA; ^6^Department of Oral Diagnostic Sciences, School of Dental Medicine, University at Buffalo, Buffalo, NY 14214, USA

**Keywords:** Temporomandibular disorder, Photobiomodulation, Monocytes, Cytokines, Inflammation

## Abstract

Temporomandibular disorders (TMD) are a prevalent source of chronic pain and disability, yet current therapies often provide limited relief with notable side effects. Photobiomodulation (PBM) has emerged as a promising non-pharmacologic approach to modulate inflammation and pain. This study investigated the effects of a dual-wavelength PBM protocol (laser + light-emitting diode (LED)) on the lipopolysaccharide (LPS)-stimulated monocyte response from individuals with painful TMD. Monocytes were isolated from 16 individuals with TMD enrolled in a randomized, placebo-controlled clinical trial (NCT05916235). Cells were plated into 96-well plates and divided into Control, LPS, and LPS + PBM treatment groups. Monocytes received four alternating-day treatments with two PBM probes: a laser probe (810/660 nm, 180 J/cm^2^) and an LED probe (660/850 nm). Cytokine and chemokine levels in culture supernatants were measured via multiplexed immunoassays. To verify cell viability after PBM treatment, the 3-(4,5-dimethylthiazol-2-yl)-2,5-diphenyltetrazolium bromide (MTT) was used. Statistical analysis was conducted using one-way analysis of variance (ANOVA) with Bonferroni correction, and *p* < 0.05 was considered significant. PBM significantly reduced levels of pro-inflammatory mediators interleukin-1 beta (IL-1β) (*p* = 0.005), C-X-C motif chemokine ligand 9 (CXCL9) (*p* = 0.0042), interferon gamma–induced protein 10 (IP-10) (*p* = 0.001), and tumor necrosis factor-alpha (TNF-α) (*p* = 0.0003), while increasing expression of regulatory mediators interleukin-10 (IL-10) (*p* = 0.0009), interleukin-1 receptor antagonist (IL-1RA) (*p* = 0.004), and CC motif chemokine ligand 17 (CCL17) (*p* = 0.003).These findings suggest that the dual-wavelength PBM protocol was able to shift the monocyte immune response from a more pro-inflammatory (M1) phenotype to a more anti-inflammatory, tissue-repair (M2) cytokine profile. These results provide additional evidence for PBM as a benign and non-invasive technique to control and potentially modify TMD-related inflammation. ClinicalTrials.gov ID: NCT05916235.

## 1. Introduction

Temporomandibular disorders (TMD) affect up to 39 million Americans, with over 10 million experiencing chronic pain [1]; the most prevalent TMDs are those with pain as the predominant characteristic. In 2001, TMDs were responsible for 17.8 million lost working days per 100 million working adults, costing billions of dollars [2]. As the second most common musculoskeletal condition after chronic low back pain, painful TMDs are characterized by pain, limited jaw function, and disability; however, current treatments such as intraoral appliances and pain medications, including opioids, provide suboptimal pain control and often cause significant side effects. Consequently, as the National Academies of Sciences Engineering and Medicine (NASEM) [3] highlighted, developing safe and effective therapies is crucial to improving the quality of life of patients with painful TMDs.

Recently, we have witnessed growing interest in Photobiomodulation (PBM) therapy due to its potential to modulate pain and inflammation. PBM, using laser and LED devices, employs low level energy to stimulate receptive cells in the body, altering gene expression, metabolism, and cytokine production [4, 5, 6]. While laser systems deliver high-intensity, focused light for deep tissue penetration, LEDs provide lower irradiance over a larger area, which may better influence inflammation, blood flow, and lymphatic drainage. The simultaneous use of both modalities is a relatively new approach with largely unexplored effects on inflammatory responses [7, 8, 9, 10]. Therefore, the objective of this study was to evaluate, *in vitro*, the impact of a dual PBM approach on the LPS-induced immune response of monocytes from individuals with painful TMD, specifically assessing its ability to modulate pro- and anti-inflammatory cytokine production.

## 2. Material and methods

Peripheral blood was collected from 16 participants (n = 16) enrolled in an ongoing double-blind, randomized, placebo-controlled clinical trial (ClinicalTrials.gov ID: NCT05916235). The protocol was approved by the University of Florida Institutional Review Board (IRB #202300986); all participants gave written informed consent specifically authorizing study participation and blood collection. A trained and certified phlebotomist collected venous blood during the participants’ baseline clinical visit. Immediately following collection, the samples were processed to isolate peripheral blood monocytes using standardized laboratory protocol described below. Isolated monocytes were then aliquoted and stored at −80 °C until used for experimental analysis. Participants’ eligibility was determined via a computer-assisted telephone interview (CATI), requiring participants to report facial pain for at least three months with average pain intensity of ≥30 (0–100 scale) during the week preceding screening. Eligible participants underwent a baseline clinical examination. A calibrated examiner (κ = 0.85) performed the Diagnostic Criteria for Temporomandibular Disorders (DC/TMD) examination to diagnose myalgia in each participant [11]. Myalgia was defined as self-reported pain in at least one masticatory muscle (masseter or temporalis, on either side), with at least one tender point eliciting familiar pain on palpation or during functional movement. Arthralgia was defined as familiar pain in one or both temporomandibular joints elicited by palpation or during joint function. “Familiar pain” refers to pain similar to what the participant has experienced over the previous 30 days.

Participants who met the diagnostic criteria for myalgia, with or without arthralgia, were instructed to complete an electronic Daily Symptom Diary (DSD) for a minimum of one week. Only those who reported an average pain score of ≥30 during that period were enrolled in the study. A baseline blood draw was performed prior to the randomization. Blood samples were collected from February 2024 to January 2025. Because appointments were scheduled throughout the day, collection times ranged from 08:00 to 17:00. All samples were processed within one hour of venipuncture.

To assess the sensitivity of human monocytes to PBM treatment, we first measured cell viability after repeated PBM exposures using the 3-(4,5-Dimethylthiazol-2-yl)-2,5-Diphenyltetrazolium Bromide (MTT) assay. For this *in vitro* protocol, a pilot test was conducted to determine the maximum number of PBM treatments that preserved cell viability. Cultures maintained 100% viability for up to four sessions, but monocyte viability declined thereafter. Accordingly, the protocol was limited to four PBM exposures to maintain cellular integrity and ensure reliable immunological assessments.

The PBM was delivered using the THOR® laser system (LX2.3, THOR Photomedicine Ltd, Chesham, Buckinghamshire, UK), which includes two active treatment probes tailored for different tissue depths. Probe A is a laser cluster comprising five laser diodes, each emitting at a wavelength of 810 nm. It delivers an irradiance of 2 W/cm^2^, applied for 30 seconds, resulting in an energy density of 60 J/cm^2^ per beam. This probe is designed for treating larger areas and targeting deeper tissues (greater than 1 cm beneath the surface). Probe B is an LED cluster consisting of 56 LEDs at 660 nm and 48 LEDs at 850 nm. It delivers an average irradiance of 50 mW/cm^2^ and is applied for 60 seconds, yielding a total energy density of 3 J/cm^2^ across the entire cluster. This probe is optimized for treating superficial inflammation, where the target tissue lies less than 1 cm below the skin.

Monocytes were isolated from each participant, adjusted to 5 × 10^6^ cells/mL and 100 mL was plated into four wells of a 96-well plate to achieve 5 × 10^5^ cells per well. This format was selected to accommodate enough cells treated with both Probe A and Probe B while ensuring consistent and controlled light exposure. Standard Petri dishes were found unsuitable due to excessive cell dispersion and inadequate control of light spillovers. The plates were divided into three experimental groups: control (no treatment), LPS (lipopolysaccharide-stimulated), and LPS + PBM, with samples plated in duplicate to enhance accuracy and reproducibility. LPS was purchased from Invitrogen (batch #5970-46-01, Carlsbad, CA, USA). Following 24 hours of incubation at 37 °C for PBM treatment, LPS + PBM plates were first exposed to Probe A for 30 seconds, then transferred to Probe B for an additional 1-minute irradiation. Control and LPS groups received no PBM. Cell culture supernatant fluids were collected at 6-hour intervals, media was refreshed, and plates were placed back at 37 °C. The treatment cycle was repeated every 48 hours until four sessions were completed (Fig. 1). Multiplex modified ELISA assays were performed on supernatants to quantify cytokine production.

**Fig. 1.** Scheme of cell treatments. LPS, lipopolysaccharide-stimulated; PBM, photobiomodulation therapy (probe A and B); Tx, treatment; H, Hour.

Statistical analyses were conducted using Statistical Package for the Social Sciences (SPSS) version 23.0 (IBM Corp., Armonk, NY, USA), with data presented as box-and-whisker plots and group comparisons made by one-way ANOVA with Bonferroni’s corrections (*p* < 0.05).

## 3. Results

Sixteen participants (14 females and 2 males), aged 19–54 years (mean = 34, standard deviation (SD) = 12.2), were enrolled in the study. The cohort comprised 14 White Caucasian individuals, 1 African American, and 1 Asian. All participants met the diagnostic criteria for a painful TMD (myalgia). Over the seven days preceding the blood draw, the average pain intensity was 48.8 (SD = 16.4), with a peak of 63 (SD = 14.7). In LPS-stimulated conditions (represented by red bars), pro-inflammatory cytokines were significantly elevated compared to unstimulated cells (Fig. 2a) and significantly depleted the anti-inflammatory cytokines compared to unstimulated cells (Fig. 2b) as expected. Following a combined PBM protocol (administered every other day for four sessions), pro-inflammatory cytokines (interleukin-1beta (IL-1β) (*p* = 0.005), C-X-C motif chemokine ligand 9 (CXCL9) (*p* = 0.0042), interferon gamma-induced protein 10 (IP-10) (*p* = 0.001) and tumor necrosis factor-alpha (TNF-α) (*p* = 0.0003)) were significantly reduced compared to LPS. At the same time, anti-inflammatory mediators interleukin-10 (IL-10) (*p* = 0.0009), interleukin-1 receptor antagonist (IL-1RA) (*p* = 0.004) and C-C Motif Chemokine Ligand 17 (CCL17) (*p* = 0.003)) significantly increased compared to LPS-stimulated cells (See Fig. 2).

**Fig. 2.** Displays the concentration of various inflammatory markers as an indicator of monocytic response under the following conditions: unstimulated controls, LPS treatment, and LPS treatment in combination with PBM treatment. (a) (M1 Phenotypic production) the columns display the concentration of four key pro-inflammatory cytokines (I–IV) typical of M1 activation (IL1β, CXCL9, IP10, and TNFα). (b) (M2 Phenotypic production) the columns display the concentration of three hallmark M2 anti-inflammatory markers. The bars of the graphs shown in Fig. 2a,b represent mean and SEM (pg/mL) of monocytic response; each dot representing data from individual subjects. The white bar (unstimulated-UNSTIM) represents the response from unstimulated controls, the red bar (+LPS) represents the response from treatment with LPS, and the green bar (+ LPS + PBM) represents the response from LPS treatment + PBM treatment. *: *p* < 0.005 by ANOVA test. PBM, Photobiomodulation; SEM, standard error of the mean; RFU, Relative Fluorescence Unit; IL, interleukin; IP-10, interferon gamma-induced protein 10; LPS, lipopolysaccharide; CXCL, C-X-C Motif Chemokine Ligand 9; TNF, tumor necrosis factor.

These results suggest that PBM shifted the immune response toward a significantly more anti-inflammatory profile, decreasing pro-inflammatory cytokines while increasing anti-inflammatory cytokines.

## 4. Discussion

In this study, PBM applied to monocyte cultures from patients with painful temporomandibular disorder (TMD) significantly decreased pro-inflammatory cytokines and increased anti-inflammatory mediators.

In previous study we characterized a hyper-inflammatory response of monocytes from women with a painful TMD in response to LPS treatment (compared to individuals without a painful TMD) that exhibited significantly higher IL-6 levels compared to controls and was positively correlated with clinical pain severity [12]. Additional studies support the idea that inflammation plays a critical role in the pathophysiology of myalgia and arthralgia. Even though its Pathophysiology is still unclear and multifactorial, increasing evidence points to immune-inflammatory processes as key contributors. Elevated levels of pro-inflammatory cytokines (*e.g.*, IL-1β, IL-6, TNF-α) and expression of anti-inflammatory markers, such as IL-10, have been consistently observed in both muscle and joint-related TMD conditions [13]. In this current study, we aimed to establish the possible efficacy of PBM treatment in modulating inflammatory outcomes at the cellular level in TMD participants.

A key concept underlying the immune response of monocyte/macrophage is the M1/M2 macrophage polarization paradigm. Macrophage polarization describes how these versatile immune cells adopt distinct functional response when exposed to specific immune activators such as the lipopolysaccharide (LPS) stimulus used in our experiments [14, 15]. Classically activated M1 macrophages display robust capacity to secrete pro-inflammatory mediators such as IL-1β, CXCL9, IP-10, and TNF-α. In contrast, activated M2 macrophages emerge in the presence of type 2 cytokines (such as IL- 4 and IL-13) and adopt an anti-inflammatory/tissue repairing profile. This M2 phenotype is marked by elevated production of regulatory cytokines, including IL-10, IL-1RA, and the chemokine CCL17, along with genes involved in extracellular matrix remodeling and wound healing [16]. The M1/M2 polarization paradigm has important implications for pain, especially in inflammatory pain conditions, myalgias, and TMD. Skewing of macrophages toward an M1 phenotype is generally associated with pro-inflammatory, pain-enhancing effects, while skewing toward M2 polarization is more associated with pain-relieving outcomes [17].

Although systematic review of PBM-TMD human clinical trials (*e.g.*, Hanna *et al*. [18] 2021, Alsarhan *et al*. [19] 2022), have demonstrated significant improvements in pain relief and mandibular function, none have directly measured *in vivo* shifts in inflammatory biomarkers. Preclinical *in vivo* experiments in a carrageenan-induced TMJ inflammation model in rats showed that a single 808 nm PBM dose (100 mW, 50 J/cm^2^) markedly inhibited leukocyte chemotaxis and reduced pro-inflammatory TNF-α and IL-1β levels, while elevating anti-inflammatory IL-10 in periarticular tissues [20]. Likewise, in a murine carrageenan paw-edema model, a 2.94 J dose of 830 nm PBM substantially attenuated both edema and carrageenan-induced thermal hyperalgesia, accompanied by downregulation of local pro-inflammatory mediators [21]. To date, no clinical investigation has linked PBM therapy to direct modulation of inflammatory markers in human TMD. Addressing this gap, the present arm of our funded trial is the first to evaluate PBM’s mechanistic, anti-inflammatory action in TMD patients, although studies in larger cohorts will be required to establish PBM as a targeted immunomodulatory treatment rather than solely a symptomatic intervention.

Our findings using purified monocytes demonstrated a change from an M1phenotypic cytokine production to an M2 phenotypic cytokine production after PBM treatment. Our data showed that monocytes isolated from the TMD patients and treated by PBM was capable of significantly decrease the LPS pro-inflammatory response to levels close to unstimulated cells and significantly increase the release of anti-inflammatory cytokine after LPS stimulation to levels superior to the baseline level of unstimulated cells. This data aligns with the concepts described by Hamblin (2017) [22] suggesting that PBM can shift the M1/M2 phenotype and aligns to several other reports in the literature showing that PBM reduces pro-inflammatory mediators such as TNF-α and IL-1β while increasing anti-inflammatory cytokines like IL-10 in inflamed tissues, including the TMJ [20]. A key innovation in our approach is applying both LED and laser light sources simultaneously with dual wavelengths. Traditional PBM studies commonly use a single treatment modality such as laser or LED with a single wavelength. However, the benefit of using both modalities together is that lasers provide deep tissue penetration with high-intensity, coherent light, while LEDs deliver broader, safer, and more superficial irradiation [20]. Animal studies have demonstrated that red/NIR light not only reduces IL-6 levels but also restores IL-10 levels [22], suggesting that combined therapy more effectively activates mitochondrial pathways and downstream signaling.

Therefore, our dual-modality approach confirmed that after only four treatments of combined LED and laser therapy significantly attenuated pro-inflammatory cytokines (IL-1β, CXCL9, IP-10, TNF-α) and enhanced tissue repair mediators (IL-10, IL-1RA, CCL17). It is important to acknowledge several key limitations that temper our findings. First, this study utilized isolated monocytes in an *in vitro* design, which cannot fully recapitulate the multicellular and tissue-level interactions that occur *in vivo* during TMD pathophysiology. Further, the interpretation of M1/M2 skewing of monocytes in response to PBM treatment as a possible mechanism of action needs to be further studied. Our sample size (n = 16) was relatively small and drawn from a single clinical site, limiting the generalizability of the findings. Finally, we also did not measure local joint or muscle tissue cytokine levels, nor correlate molecular changes with clinical pain or functional endpoints. These constraints reinforce the need for larger, multicenter *in vivo* studies that integrate mechanistic biomarkers with clinical outcomes over extended follow-up periods.

In conclusion, clinically, these findings reinforce PBM’s potential as safe, non-pharmacological treatment for patients with a painful TMD, though further studies with larger sample sizes and mechanism insights are warranted.

## 5. Key findings

• A dual wavelength PBM protocol combining laser and LED light significantly modulated the inflammatory response in monocytes from individuals with a painful TMD.

• PBM reduced LPS-induced pro-inflammatory cytokines (IL-1β, CXCL9, IP-10, TNF-α) and enhanced anti-inflammatory mediators (IL-10, IL-1RA, CCL17), indicating a shift from an M1 to M2 macrophage phenotype.

• These results support the biological plausibility of PBM as a non-pharmacological strategy for modulating systemic inflammation in chronic pain conditions such as myalgia.
